# Shoulder pain and disability index: cross cultural validation and evaluation of psychometric properties of the Spanish version

**DOI:** 10.1186/s12955-015-0397-z

**Published:** 2015-12-21

**Authors:** Miguel David Membrilla-Mesa, Antonio Ignacio Cuesta-Vargas, Rocio Pozuelo-Calvo, Victor Tejero-Fernández, Lydia Martín-Martín, Manuel Arroyo-Morales

**Affiliations:** Physical Medicine and Rehabilitation Department, Section Rehabilitation and Traumatology, Hospital Virgen de las Nieves, Granada, Spain; Department of Physical Therapy, Faculty of Health Sciencies, University of Málaga, Andalucia Tech, Instituto de Investigación en Biomedica de Malaga (IBIMA), Grupo de Clinimetria (F-14), Malaga, Spain; School of Clinical Sciences, The Queensland University of Technology, Brisbane, QLD 4059 Australia; Department of Physical Therapy, University of Granada, Granada, Spain; Department of Physical Therapy, Instituto Biosanitario Granada (IBS.Granada), Instituto Mixto Universitario Deporte y Salud (iMUDS), University of Granada, Granada, Spain

**Keywords:** Shoulder, Spanish, Patient reported outcomes

## Abstract

**Background:**

The Shoulder Pain Disability Index (SPADI) is a recently published but widely used outcome measure.

**Methods:**

This study included 136 patients with shoulder disorders. SPADI was first translated and back-translated and then subjected to psychometric validation. Participants completed the Spanish versions of the SPADI, general health (SF-12), the Simple Shoulder Test (SST), Disability of Arm, Shoulder, and Hand (DASH) questionnaires and a pain intensity visual analog scale (VAS).

**Results:**

The factors explained 62.8 % of the variance, with an internal consistency of α = 0.916 and 0.860, respectively. The confirmatory factor analysis showed a Comparative Fit Index of 0.82 and a Normed Fit Index of 0.80. The Root Mean Square Error of Aproximation was 0.12. The *x*^2^ test for the 2-factor model was significant (*x*^2^ = 185.41, df = 62, *p* < 0.01). The test-retest reliability was high, with an item ranging of the interclass correlation coefficient (ICC) from 0.89 to 0.93. The ICC for the total score was 0.91 (95 % CI 0.88 to 0.94). Measurement error by minimal detectable change (MDC)_95_ was 12.2 %. In the construct validity analysis, strong positive correlations were observed between Spanish Version of the SPADI and DASH (pain: *r* = 0.80; *p* < 0.01; disability: *r* = 0.76; *p* < 0.01). Moderate positive correlations were observed between Spanish Version of the SPADI and VAS (pain: *r* = 0.67; *p* < 0.01; disability: *r* = 0.65; *p* < 0.01). Moderate negative correlations were obtained between Spanish Version of the SPADI and SST-Sp (pain: *r* = −0.71; *p* < 0.01; disability: *r* = −0.75; *p* < 0.01). However, pain total Spanish Version of the SPADI was only weakly correlated with physical and mental components of SF-12 (both *r* = 0.40; *p* < 0.01).

**Conclusions:**

This Spanish version of SPADI demonstrated satisfactory psychometric properties in a patient sample in the hospital setting.

**Electronic supplementary material:**

The online version of this article (doi:10.1186/s12955-015-0397-z) contains supplementary material, which is available to authorized users.

## Background

Health related patient reported outcome measures (PROMs) is considered essential to determine the impact of disease on the life of individuals, taking account not only of the clinical diagnosis of a disease but also of its impact [1].

Shoulder pain is one of the most common causes of musculoskeletal pain, with a prevalence of 20–33 % in the general population [2]. Shoulder disorders are responsible for major medical, social, and economic costs [3]. They are often accompanied by pain and restricted movement, hampering certain activities [4] and compromising psychological and social wellbeing [5].

Various questionnaires are available in English to evaluate the impact of shoulder disorders on function. With regard to other languages, it is recommended to translate, culturally adapt, and validate existing instruments in order to avoid the further proliferation of different methods [6–8].

The original version of the Shoulder Pain Disability Index (SPADI) is a quality of life questionnaire developed to evaluate the pain and disability associated with shoulder dysfunction [9]. The SPADI is a 13-item shoulder function index on the ability of responders to carry out basic activities of daily living. Each item is scored by a numeric rating scale that ranges from 0 (no pain/no difficulty) to 10 (worst pain imaginable/so difficult it required help). SPADI provides a pain scale (5 items; scale score range 0–50 points, expressed as percentage) and a disability scale (8 items; scale score range 0 – 80 points, expressed as percentage). The two scale scores are averaged to derive a total Spanish Version of the SPADI score (0–100 points). A higher score indicates greater pain-related disability [9]. The original version of the SPADI was initially proposed as a visual analog scale (VAS) and subsequently validated as a numerical scale to enable administration by telephone, obtaining reliable and valid results [10].

Previous systematic reviews have found no single questionnaire to superior to others in terms of administrative burden or measurements properties [10]. There are other validated questionnaires already available as Disability of Arm, Shoulder, and Hand (DASH) or Simple Shoulder Test (SST) but several studies have recommended SPADI also, as specific scale for the shoulder due to its easy administration and rapid completion (3–10 min), which is facilitated by the brevity of the questions and the numerical response scale used (from 0 to 100) [11–13]. Systematic reviews have described SPADI as one of the highest-quality questionnaires related to the upper extremity and have endorsed its utilization [10, 13].

Although the SPADI questionnaire has been validated in German [14], Slovenian [15], Turkish [16], Italian [17], Portuguese [18], Persian [19], and Danish [20], it has not yet been validated in Spanish.

The objective of this study was to report the procedure followed for the cross-cultural adaptation and subsequent validation of a Spanish version of SPADI, including an examination of its psychometric properties.

## Methods

### Design

A two-stage observational study was conducted. The first stage comprised the translation and cross-cultural adaptation of SPADI, while the second stage consisted of a prospective evaluation of the internal consistency, reliability, construct validity and measurement error of Spanish Version of the SPADI.

#### Stage 1- Translation and cross cultural adaptation

Two physicians, with adequate expertise in shoulder disorders management and both lenguages, and an independent native professional interpreter translated the English version of SPADI into Spanish and organized a meeting to take account of possible cultural issues. A back-translation process was carried out by a specialist translator to guarantee the conceptual equivalence of the terms used, as recommended in the literature [21, 22]. People involved in translation worked independently. In a second meeting, we compared the two versions and found no appreciable differences between them. A final version of the Spanish Version of the SPADI was agreed and tested in a pilot study with 40 patients (24 females, age = 45,6 ± 13.0 years) with shoulder problems (fractures and tendinopaties) recruited from among rehabilitation outpatients at hospital setting. This pilot study included cognitive debriefing standardised interviews carried out for one member of the research staff to assess its comprehensibility and ensure that the items retained the meaning of the original version.

#### Stage 2- Evaluation of psychometric properties

##### Participants and procedure

This questionnaire validation study included 136 volunteers with different shoulder disorders recruited from among rehabilitation outpatients in a hospital setting. Inclusion criteria were the presence of a shoulder disorder and the availability of a diagnosis by a specialist rehabilitation physician; diagnoses were classified into six subcategories (Table 1). Exclusion criteria were age under 18 years and inadequate command of Spanish to complete the questionnaires. All patients signed their informed consent to participation in the study, which was approved by the Research Ethics Committee of our hospital.

**Table 1:** Demographic characteristics of the study population and the distribution of diagnoses

Characteristic	Cases (%)	Age (years) Mean (sd)
Study Population	136	49.8 ± 15.0
Male	61 (44.9 %)	46.8 ± 15.3
Female	75 (55.1 %)	52.2 ± 14.4
Diagnosis		
Humerus Fractures	29 (21.3 %)	
Calcific Tendinopathy	16 (11.8 %)	
Rotator cuff tear	24 (17.6 %)	
Osteoarthritis	3 (2.2 %)	
Hemiplegic Shoulder Pain	2 (1.5 %)	
Biceps Tendinopathy	30 (22.1 %)	
Frozen shoulder	10 (7.4 %)	
Complex Regional Pain Syndrome	4 (2.9 %)	
Avascular Necrosis	3 (2.2 %)	
Glenohumeral Instability	9 (6.6 %)	
Others^a^	6 (4.4 %)	
Questionnaires Scores Mean (SD)		
Shoulder Pain Disability Index	58.5 (22.2)	
Mental Health SF-12	38.2 (9.0)	
Physical Health SF-12	47.5 (11.0)	
Simple Shoulder Test	34.5 (24.14)	
Disability of Arm, Shoulder and Hand	48.1 (20.4)	
Visual Analogue Scale	4.0 (2.6)	

^a^Minor-Heterogeneous shoulder disorders: Polymyalgia reumathic (2), Acromioclavicular Luxation (2), Unspecific Shoulder Pain (2)

Participants who met the selection criteria completed a Spanish version of the SF-12 [23], SST [24], DASH [25] questionnaires and VAS in the hospital with the assistance of rehabilitation service staff. SF-12 (Version 1) is a self-administered instrument with 12 items on physical and mental health status; responses are scored (for intensity or frequency) on a Likert-type scale (3–6 points according to the item). These items are used to calculate the physical and mental summary measures. This instrument has shown adequate reliability (ICC = 0.73-0.86) [23]. SF12 was used to check discriminant construct validity. The SST is a 12-item shoulder function scale on the ability (yes/no) of respondents to perform 12 activities of daily living (ADLs). The total SST score (0 to 100) expresses the percentage of items with a positive response. The Spanish version of the SST was recently validated and showed adequate reliability (ICC = 0.69–0.94) [24]. The 30-item DASH measures the function and symptoms of patients with upper extremity musculoskeletal disorders. The total score ranges from 0 (best state) to 100 (worst). The Spanish version of DASH has been validated and showed adequate reliability (Cronbach alpha = 0.96) [25]. SST and DASH were usded to check convergent validity. Pain intensity was tested using a VAS (0 to 10).

A randomly selected subgroup (*n* = 56) of the total sample repeated the questionnaires after an interval of 24–48 h to study the reliability of the Spanish Version of the SPADI using a test-retest methodology. This sample was selected using a random numeric sequence generated by a computer. A brief interval of 24–48 h was selected to avoid fluctuations in the severity of the symptoms. The questionnaires were administrated in same conditions used in the all sample.

### Statistical analysis

Sample size was selected in accordance with recommendations to include 4–10 subjects per variable, with a minimum sample size of 100 subjects to ensure stability of the variance–covariance matrix in the confirmative factor analysis [26].

Means and standard deviations of the demographic variables were calculated. Construct validity and factor structure were then determined from maximum likelihood extraction (MLE) with varimax rotation, establishing the satisfaction of the following three criteria as a priori extraction requirement: scree-plot inflection, eigenvalue >1.0, and variance >10 %. The fit of confirmatory factor analysis was considered to be acceptable if the comparative fit index (CFI) and the normalized fit index (NFI) were greater than 0.90, with root mean square error of approximation (RMSEA) values equal to or less than 0.08 [27, 28] The internal consistency of measures was evaluated by determining the Cronbach alpha coefficient in an expected range of 0.70 to 0.90 [29]. The test-retest reliability was analyzed by using the type 2.1 interclass correlation coefficient, and the error sensitivity was calculated with the MDC95 analysis of Stratford. Where MDC = 1.96 × SEM × square root of 2 [30]. The construct validity was determined by comparing Spanish Version of the SPADI with SF-12, SST, DASH and VAS scores. Correlations were calculated using Pearson approach. A correlation value below 0.25 indicates a weak relationship, a value between 0.25 and 0.50 a fair relationship, a value between 0.50 and 0.75 a moderate to strong relationship, and a correlation above 0.75 a strong relationship [31]. Moderate to strong positive (DASH, VAS) and negative (SST) correlations with Spanish Version of the SPADI were expected, with a similar pattern of expected correlations for both dimensions of the index. A lower correlation was expected between Spanish Version of the SPADI and SF-12, which is a generic functionality scale. SPSS version 21.0 for IOS (IBM, Chicago, IL) and LISREL v.8.8 (SSI Inc., Lincolnwood, IL) were used for the statistical analysis.

## Results

Potentially eligibles participants in database during the period of the study were 234 patients wich were invited for eligibilty 172 patients. 36 patients refused to participate and finally 136 participants were included. 40 participants completed the second questionnaire of the 56 invited. All participants comprehend the questionnaire and found it easy to complete. No conceptual ambiguities or language difficulties were encountered in the translation of the SPADI (Additional file 1). Minor changes in the Spanish version included the replacement of imperial with metric measures, e.g., “10 lb” becomes “4.5 Kg” in item 12. After the cognitive debriefing interviews ensured that the items retained the meaning of the original version.

**Fig. 1:**
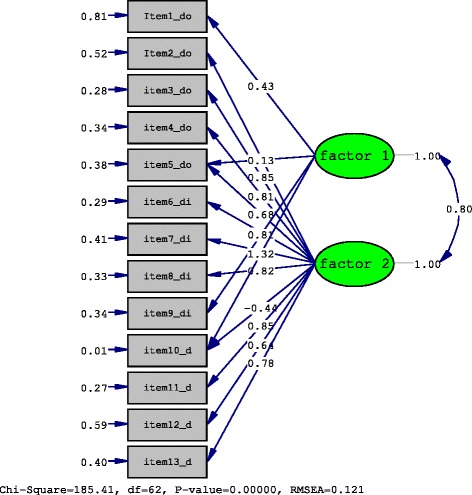
The *x*2 test for the 2-factor model was significant

Table 1 exhibits the demographic characteristics of the participants and the distribution of diagnoses. In the factorial analysis, the correlation matrix for Spanish Version of the SPADI was adequate according to results of the Kaiser-Meyer-Oklin (0.92) and Bartlett’s sphericity test (*p* < 0.001). A 62.8 % of the variance was explained by two factors. The item loading is shown in Table 2: factor 2 comprised items 1, 9, and 10, while factor 1 included the remaining items with the exception of item 13, which showed cross-loading. The confirmatory factor analysis showed a Comparative Fit Index of 0.82 and a Normed Fit Index of 0.80. The Root Mean Square Error of Aproximation was 0.12. The *x*2 test for the 2-factor model was significant (*x*^2^ = 185.41, df = 62, *p* < 0.01 (Fig. 1).

**Table 2:** Factor loading items for the two-factor solution

	Component	
	1	2
At its worst?	,22	**,55**
When lying on the involved side?	**,63**	,36
Reaching for something on a high shelf?	**,81**	,32
Touching the back of your neck?	**,70**	,44
Pushing with the involves arm?	**,68**	,44
Washing your hair?	**,83**	,25
Washing your back?	**,82**	,17
Putting on an undershirt or pullover sweater?	**,67**	,52
Putting on a shirt that buttons down the front?	,28	**,85**
Putting on your pants?	,24	**,88**
Placing an object on a high shelf?	**,87**	,22
Carrying a heavy object of 10 lb?	**,57**	,40
Removing something from your back pocket?^a^	**,61**	**,56**

^a^Cross loading

The bold numbers represent the main factor load in each component

For the Spanish Version of the SPADI, there were no significant missing responses (<7 missing response in 136 patients), similar level of missing response was found in the rest of questionnaires used in this study. After check the origen of this missing data a missing at random was assumed. A high degree of internal consistency was obtained for each factor α = 0.92 (CI95 % 0.91 to 0.95) and 0.82 (CI95 % 0.76 to 0.86), The test-retest reliability was also evaluated with ICC, with an item ranging from 0.89 to 0.93. The ICC for the total score was 0.91 (95 % CI 0.88 to 0.94). Measurement error by MDC_95_ was 12.2 %.

The construct validity was confirmed by strong positive correlations between the Spanish Version of the SPADI and DASH (pain: *r* = 0.80; *p* < 0.01; disability: *r* = 0.76; *p* < 0.01), moderate positive correlations between SPADI and VAS (pain: *r* = 0.67; *p* < 0.01; disability: *r* = 0.65; *p* < 0.01), moderate negative correlations between SPADI and SST (pain: *r* = −0.71; *p* < 0.01; disability: *r* = −0.75; *p* < 0.01) and weak positive correlation with physical and mental components of SF-12 (both *r* = 0.40; *p* < 0.01).

## Discussion

SPADI demonstrated good internal consistency and convergent validity and reliability in a sample of 136 patients with different shoulder disorders.

The confirmatory factor analysis showed an acceptable fit with a CFI of 0.82 and NFI of 0.80, but the error was higher (RMSEA = 0.12) than the recommended value of 0.08 [28]. The internal consistency value calculated for this version was similar to that obtained for versions validated in other languages [14–20] and within an acceptable range, in common with the original questionnaire and all other published versions [26]. The Spanish version showed a clear difference in the loading of the two factors, with items that evidenced a high correlation with one of the factors and a low correlation with the other. Our finding contrasts with the similar loading of the two factors reported for the original questionnaire [9] and subsequent versions [32], which hindered a clear separation between pain and disability dimensions. The fit indices associated with the confirmatory factor analysis model were satisfactory, although the error of approximation was an exception and did not indicate an optimal fit, which may possibly be due to an effect of our specific study population on the factor structure. It should be borne in mind that a slightly increased error does not necessarily imply that the structure of the scale is poor. The good construct validity obtained with this Spanish version supports its usefulness for evaluating patients’ perception of the impact of shoulder lesions.

The test-retest reliability of Spanish Version of the SPADI (0.89–0.93) was superior to that reported for the original questionnaire (0.64–0.66), slightly higher than that of the Persian [19] and Danish [20] versions, and similar to that of the Brazilian [18], German [14], Turkish [16], and Slovenian [15] versions. Perhaps, the reason could be a Spanish sample is more homogenous. The Measurement error by MDC_95_ was 12.2 %, lower than the original version, was of 18 % [11].

As expected, the lowest correlation (divergent construct validity) was observed between SPADI and SF-12, reflecting the discriminative validity of the instrument, given that SF-12 is a generic functionality scale. Moderate-strong correlations were found with the specific shoulder-related instruments (SST and DASH), indicating adequate convergent validity. Low-moderate correlation was found between SPADI and VAS, as observed in previous cross-cultural validation studies [14, 20].

The present study population was limited to hospital outpatients in a Spanish urban setting, and different results may be obtained in other types of population. The greater reliability observed for the Spanish version than for the original questionnaire may be attributable to the shorter interval between tests (48 h) which could induce an artificial inflation of correlation coefficients due to recall bias. Studies of this version of the questionnaire are warranted to test its validity in other Spanish-speaking countries (e.g., Latin America, Philippines, etc.). A study strength is that the sample size was larger (*n* = 136) than in previous validation studies of this instrument, reducing standard errors and supporting its application in individual and group studies [33]. Further research is needed to establish longitudinal validity, responsiveness and thresholds for minimal important change in the Spanish version of SPADI.

## Conclusions

In conclusion, the psychometric properties of the Spanish version of SPADI are similar to those of the original questionnaire and subsequent adaptations in different languages, supporting its utilization as a reliable clinimetric instrument in the setting of shoulder disorders.

## Additional file

Additional file 1:
**Shoulder and Pain Disability Index (SPADI-Sp).** (PDF 60 kb)

## References

[1] World Health Organization (2001). ICF - International Classification of Functioning, Disability and Health.

[2] McBeth J, Jones K (2007). Epidemiology of chronic musculoskeletal pain. Best Pract Res Clin Rheumatol.

[3] Virta L, Joranger P, Brox JI, Eriksson R (2012). Costs of shoulder pain and resource use in primary health care: a cost-of-illness study in Sweden. BMC Musculoskelet Disord.

[4] Largacha M, Parsons IM, Campbell B, Titelman RM, Smith KL, Matsen F (2006). Deficits in shoulder function and general health associated with sixteen common shoulder diagnoses: a study of 2674 patients. J Shoulder Elbow Surg.

[5] Paananen M, Taimela S, Auvinen J, Tammelin T, Zitting P, Karppinen J (2011). Impact of self-reported musculoskeletal pain on health- related quality of life among young adults. Pain Medicine.

[6] Beaton DE, Bombardier C, Guillemin F, Ferraz MB (2000). Guidelines for the process of cross-cultural adaptation of self-report measures. Spine (Phil 1976).

[7] Kirkley A, Alvarez C, Griffin S (2003). The development and evaluation of a disease-specific quality-of-life questionnaire for disorders of the rotator cuff: The Western Ontario Rotator Cuff Index. Clin J Sport Med.

[8] Leggin BG, Michener LA, Shaffer MA, Brenneman SK, Iannotti JP, Williams GR (2006). The Penn shoulder score: reliability and validity. J Orthop Sports Phys Ther.

[9] Roach KE, Budiman-Mak E, Songsiridej N, Lertratanakul Y (1991). Development of a shoulder pain and disability index. Arthritis Care Res.

[10] Schmidt S, Ferrer M, González M, González N, Valderas JM, Alonso J (2014). Evaluation of shoulder-specific patient-reported outcome measures: a systematic and standardized comparison of available evidence. J Shoulder Elbow Surg.

[11] Williams JW, Holleman DR, Simel DL (1995). Measuring shoulder function with the Shoulder Pain and Disability Index. J Rheumatol.

[12] MacDermid JC, Solomon P, Prkachin K (2006). The Shoulder Pain and Disability Index demonstrates factor, construct and longitudinal validity. BMC Musculoskelet Disord.

[13] Bot SD, Terwee CB, van der Windt DA, Bouter LM, Dekker J, de Vet HC (2004). Clinimetric evaluation of shoulder disability questionnaires: a systematic review of the literature. Ann Rheum Dis.

[14] Angst F, Goldhahn J, Pap G, Mannion AF, Roach KE, Siebertz D (2007). Cross-cultural adaptation, reliability and validity of the German Shoulder Pain and Disability Index (SPADI). Rheumatology (Oxford).

[15] Jamnik H, Spevak MK (2008). Shoulder Pain and Disability Index: validation of Slovene version. Int J Rehabil Res.

[16] Bicer A, Ankarali H (2010). Shoulder pain and disability index: a validation study Turkish women. Sind Med J.

[17] Marchese C, Cristalli G, Pichi B, Manciocco G, Mercante G, Pellini R (2012). Italian cross-cultural adaptation and validation of three different scales for the evaluation of shoulder pain and dysfunction after neck dissection: University of California-Los Angeles (UCLA) Shoulder Scale, Shoulder Pain and Disability Index (SPADI) and Simple Shoulder Test (SST). Acta Otorhinolaryngol Ital.

[18] Martins J, Napoles B, Hoffman C, Oliveira A (2010). Versão brasileira do Shoulder Pain and Disability Index: tradução, adaptação cultural e confiabilidad. Revista Brasileira Fisioterapia São Carlos.

[19] Ebrahimzadeh MH, Biriandineiad A, Golhasani F, Moradi A, Vahedi E, Kachooei AR. Cross-cultural adaptation, validation, and reliability testing of the Shoulder Pain and Disability Index in the Persian population with shoulder problems. Int J Rehabil Res. 2013; Oct 9. [Epub ahead of print]. 10.1097/MRR.0000000000000088 25305009

[20] Christiansen DH, Andersen JH, Haahr JP (2013). Cross-cultural adaption and measurement properties of the Danish version of the Shoulder Pain and Disability Index. Clin Rehabil.

[21] Cuesta-Vargas A, Gonzalez-Sanchez M, Farasyn A (2010). Development of a Spanish version of the “Backache Index” Cross cultural linguistic adaptation and reliability. J Back Musculoskelet Rehabil.

[22] Muñiz J, Elosua P, Hambleton RK (2013). International test commission guidelines for test translation and adaptation. Psicothema.

[23] Gandek B, Ware JE, Aaronson NK, Apolone G, Bjorner JB, Brazier JE (1998). Cross-validation of item selectionand scoring for the SF-12 Health Survey in nine countries: results from the IQOLA Project International Quality of Life Assessment. J Clin Epidemiol.

[24] Membrilla-Mesa MD, Tejero-Fernández V, Cuesta-Vargas AI, Arroyo-Morales M (2014). Validation and reliability of a Spanish version of Simple Shoulder Test (SST-Sp). Qual Life Res.

[25] Hervás MT, Navarro-Collado MJ, Peiró S, Rodrigo-Pérez JL, López- Mateú P, Martínez-Tello I (2006). Spanish version of the DASH questionnaire.Cross-cultural adaptation, reliability, validity and responsiveness. Med Clin.

[26] Terwee CB, Bot SD, de Boer MR, van der Windt DA, Knol DL, Dekker J (2007). Quality criteria were proposed for measurement properties of health status questionnaires. J Clin Epidemiol.

[27] Schumacher RE, Lomax RGA (1996). Beginner's guide to structural equation modeling.

[28] Hu L, Bentler PM (1999). Cutoff criteria for fit indexes in covariance structure analysis: Conventional criteria versus new alternatives. Struct Equ Model.

[29] Mokkink LB, Terwee CB, Knol DL, Stratford PW, Alonso J, Patrick DL (2010). The COSMIN checklist for evaluating the methodological quality of studies on measurement properties: a clarification of its content. BMC Med Res Methodol.

[30] Stratford PW (2004). Getting more from the literature: Estimating the standard error of measurement from reliability studies. Physiother Can.

[31] Colton T (1974). Statistics in Medicine.

[32] Roddey TS, Olson SL, Cook KF, Gartsman GM, Hanten W (2000). 30.-Comparison of the University of California-Los Angeles Shoulder Scale and the Simple Shoulder Test with the shoulder pain and disability index: single-administration reliabilityand validity. Phys Ther.

[33] Fayers PM, Machin D (2007). Scores and measurements: validity, reliability, sensitivity. Quality of life: The assessment, analysis and interpretation of patient reported outcomes.

