# Supplementary material for: Shoulder pain and disability index: cross cultural validation and evaluation of psychometric properties of the Spanish version
Source: Health Qual Life Outcomes. 2015 Dec 21;13:200. doi: 10.1186/s12955-015-0397-z (PMC4687317; doi:10.1186/s12955-015-0397-z)
Supplement: Additional file 1: — Shoulder and Pain Disability Index (SPADI-Sp). (PDF 60 kb) [file 12955_2015_397_MOESM1_ESM.pdf]

SHOULDER AND PAIN DISABILITY INDEX (SPADI-Sp)

Por favor, ponga una cruz en el número que mejor represente su experiencia durante la última semana como consecuencia de su problema de hombro.

Escala de dolor: ¿Cómo de grave es el dolor?

Ponga una cruz en el número que mejor describa su dolor, donde: 0 = ausencia de dolor y 10 = el peor dolor imaginable

¿En su peor momento?	0	1	2	3	4	5	6	7	8	9	10
¿Cuándo se tumba sobre ese lado?	0	1	2	3	4	5	6	7	8	9	10
¿Al alcanzar algo en un estante alto?	0	1	2	3	4	5	6	7	8	9	10
¿Al tocarse la parte posterior de su cuello?	0	1	2	3	4	5	6	7	8	9	10
¿Al empujar con el brazo afecto?	0	1	2	3	4	5	6	7	8	9	10

Escala de Discapacidad: ¿Cuánta dificultad tiene usted?

Ponga una cruz en el número que mejor describa su experiencia, donde: 0 = sin dificultad y 10 = tan difícil que requiere ayuda.

¿Lavándose el pelo?	0	1	2	3	4	5	6	7	8	9	10
¿Lavándose la espalda?	0	1	2	3	4	5	6	7	8	9	10
¿Poniéndose una camiseta o un jersey?	0	1	2	3	4	5	6	7	8	9	10
¿Poniéndose una camisa con los botones delante?	0	1	2	3	4	5	6	7	8	9	10
¿Poniéndose los pantalones?	0	1	2	3	4	5	6	7	8	9	10
¿Colocando un objeto en un estante alto?	0	1	2	3	4	5	6	7	8	9	10
¿Llevando un objeto pesado (4.5 kg)?	0	1	2	3	4	5	6	7	8	9	10
¿Cogiendo algo de su bolsillo trasero?	0	1	2	3	4	5	6	7	8	9	10
